# Critical appraisal of tobacco dependence treatment guidelines

**DOI:** 10.1007/s11096-020-01110-4

**Published:** 2020-09-08

**Authors:** Maguy Saffouh El Hajj, Myriam Jaam, Saba Abdal Salam Sheikh Ali, Rana Saleh, Ahmed Awaisu, Bridget Paravattil, Kyle John Wilby

**Affiliations:** 1 College of Pharmacy, QU Health, Qatar University, 2713, Doha, Qatar; 2 Hamad Medical Corporation, Doha, Qatar; 3 School of Pharmacy, University of Otago, PO Box 56, Dunedin, 9054 New Zealand

**Keywords:** AGREE II, Critical appraisal, Guideline, Smoking cessation, Tobacco cessation

## Abstract

*Background* Tobacco use is a leading preventable cause of morbidity and mortality globally. Clinical practice guidelines for the treatment of tobacco use dependence are of varied scope and quality, making it challenging for users to select and apply recommendations. *Objective* The study objective is to identify and critically appraise the quality of existing clinical practice guidelines for tobacco cessation. *Setting* The study occurred between collaborative academic institutions located in Qatar and New Zealand. *Methods* A systematic literature search was performed for the period 2006–2018 through the following databases: PubMed, EMBASE, CINAHL, ISI Web of Science, Scopus, National Guideline Clearing House, Campbell Library, Health System Evidence, Joanna Briggs Institute Evidence-Based Practice Database, Academic Search Complete, ProQuest, PROSPERO, and Google Scholar. Relevant professional societies’ and health agencies’ websites were also searched. Two reviewers independently extracted and assessed guidelines’ quality using Appraisal of Guidelines, Research and Evaluation II (AGREE II) instrument. *Main outcome measure* Standardized domain scores according to the AGREE II instrument. *Results* 7741 hits were identified. After removing duplicates and screening, 24 guidelines were included. Highest guideline quality was for National Institute for Health and Care Excellence (NICE) guideline with an overall ranking score of 87.56% and least quality was for Japanese Circulation Society Joint Working Group with an overall score of 29.34%. Domain 4 of AGREE II (clarity of presentation) had the highest average quality score (70.95%), while the lowest average quality scores were for Domain 2 (Rigour of Development) (50.21%) and Domain 5 (Applicability) (45.05%). *Conclusion* Seven guidelines were judged to be of high quality (overall score of ≥ 70%). Future guidelines for tobacco dependence treatment should use rigorous methods of development and provide applicable recommendations.

**Electronic supplementary material:**

The online version of this article (10.1007/s11096-020-01110-4) contains supplementary material, which is available to authorized users.

## Impact of findings on practice

- The study identified 25 eligible tobacco cessation Clinical Practice Guidelines (CPGs) of which seven guidelines were considered of high quality.
- In order to improve the application of the best evidence-based recommendations in clinical practice, future tobacco cessation guidelines developers should apply robust methodologies based on quality criteria such as AGREE II criteria in developing and reporting the guidelines.
- Before adaptation in national practice, clinicians should rigorously appraise the quality of CPGs and assess their applicability to their context taking into consideration several factors including cultural, financial, health system and environmental factors

## Introduction

Tobacco use and dependence is a leading preventable cause of morbidity and mortality globally, and is strongly linked to numerous diseases and healthcare burden [1]. Worldwide, the age standardized prevalence of daily smoking was 25% for men and 5.4% for women from 1990 to 2015 [2]. Although concerted efforts have been put in place to reduce the global smoking prevalence, many countries continue to record high smoking rates, resulting in increased disease burden and healthcare expenditures [2, 3]. Tobacco cessation interventions play an important role in addressing tobacco-related health risks and mortality. Studies have shown that when pharmacotherapy and behavioral interventions are used among adults either singly or in combination, this results in higher success rates [4–7]. Therefore, the role for healthcare providers in implementing tobacco cessation interventions within their clinical practice settings becomes unequivocally important. Clinicians use tobacco cessation tools and resources such as clinical practice guidelines (CPGs) to provide tobacco use dependence management.

CPGs are important resources used by clinicians when making evidence-based decisions to improve the quality and outcomes of the care delivered to patients. CPGs are attractive due to their concise nature and capacity to provide consistent care by clinicians [8]. On the other hand, CPGs have their limitations when recommendations are influenced by expert opinion and practice experiences. In addition, CPGs are adopted into patient care without undergoing robust critical appraisal to assess their validity and methodological quality [9]. Evidence-based CPGs for the treatment of tobacco use dependence are of varied scope and quality, making it challenging for clinicians to select and apply the best evidence-based recommendations. Having high quality critically appraised tobacco cessation guidelines is important for clinicians to apply the best evidence when treating their patients for tobacco use dependence.

## Aim of the study

This study aimed to critically appraise current tobacco cessation guidelines and to determine the ones with the highest quality for potential utilization in clinical practice.

## Ethics approval

No approval was necessary.

## Method

### Search strategy and identification of guidelines

A protocol for the systematic review was developed using the Preferred Reporting Items for Systematic Reviews and Meta-Analyses (PRISMA) guidelines [10] and other best practices. The protocol was registered and published on PROSPERO at the Centre for Reviews and Dissemination, UK (CRD42018086709). We conducted a systematic literature search to identify tobacco cessation CPGs. The following electronic databases were searched to identify eligible articles published from January 2006 to June 2018: PubMed, EMBASE, CINAHL, ISI Web of Science, Scopus, National Guideline Clearing House, Campbell Library, Health System Evidence, Joanna Briggs Institute Evidence-Based Practice Database, Academic Search Complete, ProQuest, PROSPERO, and Google Scholar. In order to retrieve relevant guidelines, search terms were combined from four different categories using Boolean operators (Table 1). The keywords were customized to each database specific indexing terms such MeSH terms in PubMed.

**Table 1:** Search Terms

Category	Search terms
Category A	Tobacco-smoking-cigarette-shisha-nicotine
Category B	Treatment-management-strategy-intervention-pharmacological-behavioral-diagnos*-care-evaluation-assessment-therapeutic-counseling-behavior-psychotherapy-electronic cigarette-motivational-advise-interview-cognitive-psychosocial-service
Category C	Nicotine-varenicline-bupropion-clonidine-nortriptyline
Category D	Tobacco use-dependence-cessation-addiction-abstinence-quit-relapse-stop-harm reduction
Category E	Guideline-guidance-CPG-consensus-opinion-recommendation-policy-summary-statement-position-practice-bulletin-procedure-protocol

Other resources identified such as relevant articles were manually reviewed to further identify additional guidelines not found in the electronic searches. In addition, relevant guidelines’, professional societies’ (cancer, cardiovascular, lung diseases, tobacco and substance abuse, addiction), and government agencies’ websites were searched for relevant tobacco cessation guidelines. These include: National Guideline Clearing House; National Institute for Health and Clinical Excellence (NICE); World Health Organization (WHO); US Center for Disease Control (CDC); US Department of Health and Human Service (USDHHS); clearing houses of Australia, New Zealand, Canada, the United States and the United Kingdom; International Union Against Tuberculosis and Lung Disease (IUATLD); smoking cessation guidelines of cancer, cardiovascular, and respiratory diseases [examples include National Comprehensive Cancer Network (NCCN guidelines)]. All search results were imported into EndNote^®^ X7 reference management software. Each electronic database or website was assigned to two of the study investigators who independently searched the database/website and combined the search results. Duplicates were removed prior to screening.

### Eligibility criteria and guidelines selection

CPGs were eligible for inclusion if they provide recommendations for the treatment of tobacco use dependence (pharmacologic and/or non-pharmacologic). We included only the most recent version of each available CPG published from 2006 to 2018. Furthermore, guidelines targeting specific populations (e.g. tuberculosis, pregnant women, COPD) were included in the review if they were exclusively about tobacco dependence treatment in the specific sub-populations. Guidelines for related conditions such as asthma, COPD, cardiovascular diseases, and tuberculosis which contain tobacco use treatment or smoking cessation as part of the guidelines (e.g. a section on tobacco dependence treatment within the guideline) were excluded from the review. In addition, guidelines were excluded if they were non-English, non-peer reviewed, or published prior to 2006 without full updates within 2006–2018. Guidelines were also excluded if recommendations were provided with no level of evidence or no grades of recommendations assigned to them. Reviews, letters, editorials, and commentaries about published CPGs were also excluded. However, executive summaries and other supplementary documents were marked as supporting resources for any additional relevant information during data extraction.

Titles and abstracts from the electronic searches were independently screened by two reviewers for potential eligibility using the above predefined eligibility criteria. Furthermore, two independent reviewers read the full-text of each CPG identified from the title/abstract screening for inclusion in the review. Discrepancies between the reviewers were resolved through discussion at all stages of search and screening process. In case of non-consensus, a third reviewer was consulted.

### Data extraction

We extracted information related to the characteristics of each CPG document including the publisher, authors, year of publication, funding source, organization involved in the CPG development, target population and/or subpopulations, and the guidelines development methodology. To increase the validity and consistency of the extracted data, two reviewers independently extracted the information and any discrepancies were resolved through discussion.

### Critical appraisal and quality assessment of the included guidelines

The quality of each of the included tobacco cessation CPGs was assessed by two independent reviewers. Seven reviewers conducted the quality appraisals. The CPGs were assessed using the AGREE II (Appraisal of Guidelines, Research and Evaluation II) instrument [11]. This instrument is widely used for CPGs development, reporting, and evaluation. It contains six constructs with 23 evaluation criteria graded on a seven-point Likert type scale (1 = strongly disagree to 7 = strongly agree). The six constructs of the AGREE II include: (1) scope and purpose, (2) stakeholder involvement, (3) rigor of development, (4) clarity and presentation, (5) applicability and, (6) editorial independence. All reviewers read the AGREE II User Manual to standardize and guide the appraisal process. In addition, several of the reviewers had familiarity and experience with the use of the AGREE II instrument. One investigator (KW) had previously published studies using the AGREE II instrument and was available for consult by other reviewers, if needed. All investigators had clinical and/or research experience in the field of tobacco dependence and its treatment.

Each CPG was appraised by two independent assessors using the AGREE II instrument. Scores were assigned for each of the 23 criteria in a shared Excel spread sheet. The project leader (MH) collected and combined all the assessments in another master Excel spreadsheet. Weighted domain scores were calculated as described in the AGREE II User Manual using Microsoft Excel. Average domain scores for each guideline were also calculated. In addition to the items assessment, for each CPG, each of the two assessors judged the CPG as recommended, recommended with modifications, or not recommended (as per AGREE II criteria). In case of any disagreement in the endorsements, the two raters discussed in a face-to-face meeting and resolved the discrepancies through consensus or adjudication with a third reviewer. Agreement was calculated between the two reviewers for the appraised items of each guideline using two-way random (absolute agreement) Intraclass Correlation Coefficient (ICC). The ICC scores and 95% confidence intervals were calculated using IBM SPSS statistics software version 22.

## Results

**Fig. 1:**
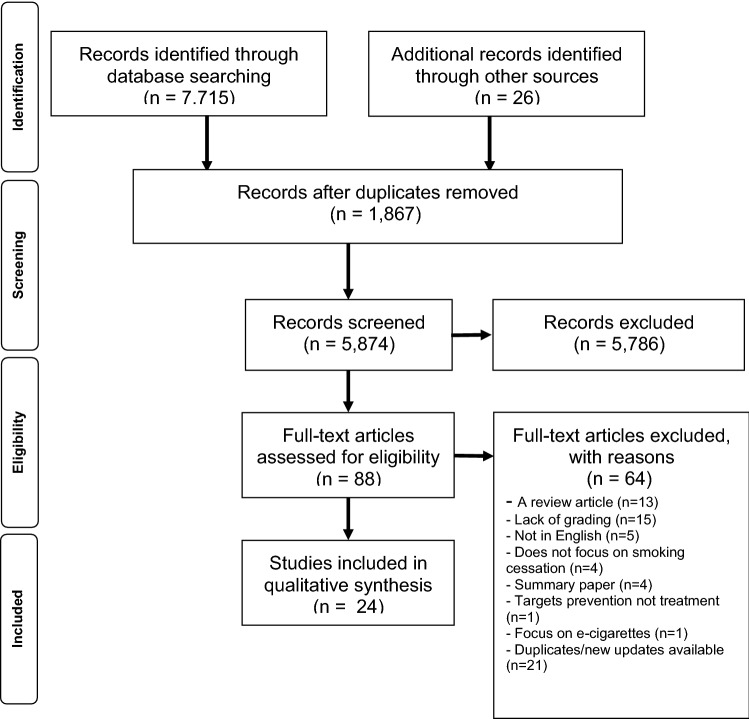
Articles flow diagram

Following the search of the databases, 7715 records were identified in addition to 26 records identified through organizational websites and systematic review references. After removing duplicates and screening (titles, abstracts, full-texts), a total of 24 guidelines related to tobacco cessation that satisfied the study eligibility criteria were identified and evaluated (Fig. 1).

Table 2 describes the characteristics of the CPGs included in the review. The 24 guidelines developed by a wide range of organizations (from governmental to professional bodies) were published between 2006 and 2018. Different sources of guideline-development funding were reported; some guidelines were sponsored by pharmaceutical companies, while others received governmental funding [12–19]. Two guidelines were sponsored by Pfizer, one guideline by GalaxoSmithKline, and one guideline by Boehringer Ingelheim Pharmaceuticals Inc as unrestricted grants while many did not report the funding source [17, 20–29]. All the included guidelines were targeted to healthcare providers and focused on patients who are tobacco smokers. Some guidelines included recommendations related to subpopulations including: children and teenagers (14 guidelines) [13, 14, 16, 17, 19, 22, 24–26, 28, 30–33]; patients with mental illnesses (12 guidelines) [13, 14, 16, 17, 19, 24, 25, 28, 30–33]; and pregnant women (13 guidelines) [13, 14, 16, 17, 19, 24–26, 28, 31–34]. Moreover, four guidelines covered patients undergoing surgical interventions as a subpopulation [17, 22, 30, 33], while two guidelines included prisoners [14, 25].

**Table 2:** Characteristics of included tobacco dependence treatment guidelines

Author/year	Publisher	Funding source	Main targeted population	Targeted subpopulations	Search strategy	Resources of literature	Language restriction	Dates of search
Weel/2006 [30]	The programme Evidence-Based Guideline Development of the Dutch Society of Medical Specialists, Dutch Institute for Healthcare	Partnership on smoking cessation and Dutch Society of Medical Specialists	All smokers	Parents of newborns and young children; teenagers; patients undergoing a surgical intervention; mental illnesses; multiple addiction	Systematic review	**3 databases** (Medline, CDSR, EMBASE)	NR	Inception – 2003
Tønnesen et al./2007 [20]	European Respiratory Society Task Force	NR	Respiratory diseases (COPD/lung cancer)	NA	Meta-analyses of 6000 studies	NR	NR	NR
Fiore et al./2008 [35]	U.S. Public Health Service	Multiple organizations and academic institutions	All adult smokers	NR	Meta-analyses	**11 databases** (NR)	English	1999–2007
Reis et al./2008 [12]	Center for evidence based medicine – University of Lisbon School of Medicine	Unrestricted grant from Pfizer	All adults Smokers	NA	Systematic review	**6 databases** (PubMed; Cochrane Central Register of Controlled Trials; CDSR; DARE; EMBASE; CINAHL) and organizations’ websites	Portugues, French, English	Inception – 2007
Schayck et al./2008 [13]	International Primary Care Respiratory Group (IPCRG)	Unrestricted educational grant from Pfizer Inc (Europe)	All adult smokers	Pregnant women; adolescents; mental illness	Available reviews and guidelines were reviewed	NR	NR	NR
Kelly et al./2010 [21]	National Institute for Health and Care Excellence (NICE)	NR	Women who are pregnant or planning a pregnancy, or who have a child aged up to 12 months and their families and carers	NA	Systematic review	**11 databases** (ASSIA; British Nursing Index; CINAHL; EMBASE; Maternity and Infant Care; PsychINFO; Science Citation Index; Social Science Citation Index; Web of Science; Google Scholar)	English	1990 – 2009
Zwar et al./2011 [14]	Royal Australian College of General Practitioners	Educational grant by Glaxo Smith Kline (GSK) Australia	All adults smoking	Aboriginal and Torres Strait Islander people; culturally and linguistically diverse groups; pregnant and lactating women; adolescents and young people; mental illness; substance use problems; prisoners; people with smoking related diseases	NR	NR	NR	NR
Leone F et al./2012 [15]	The American College of Chest Physicians	American College of Chest Physicians. The lung cancer guidelines, Lung Cancer Research Foundation. And educational grant from Boehringer Ingelheim Pharmaceuticals, Inc	Patients with lung cancer who use tobacco	NA	Systematic review	**6 databases** (MEDLINE, EMBASE, CINAHL, PsychINFO, the Cochrane Collaborative, Google Scholar)	English	1985 – present
Lingford-Hughes et al./2012 [16]	British Association for Psychopharmacology	Multiple pharmaceutical companies	People with substance abuse or harmful use or addiction and with psychiatric comorbidity	Pregnancy; mental illnesses; children and adolescents; older adults (above 65 years old)	Systematic search and consensus	**3 databases** (MEDLINE, EMBASE, CDSR) and experts	NR	NR
Murohara et al./2012 [22]	Japanese Circulation Society (JCS) Joint Working Group	NR	All adults who smoke	cardiovascular diseases; respiratory diseases; women; children and adolescents; dental and oral cavity disorders; status before surgery and surgical diseases	NR	NR	NR	NR
Selby et al./2012 [31]	Canadian Action Network for the Advancement, Dissemination and Adoption of Practice-informed Tobacco Treatment (CAN-ADAPTT)	Drugs and Tobacco Initiatives Program, Health Canada	All adults tobacco users	Aboriginal peoples; hospital-based populations; mental health and/or other addiction(s); pregnant and breastfeeding women; youth (children and adolescents)	Systematic search	**3 databases** (NR) and organizations’ websites	English	NR
Chan K et al./2013 [17]	Health Promotion Board of Singapore	NR	All tobacco users	Hospitalized and pre-operative patients; adolescents; pregnancy; mental health; cardiovascular disease; alcohol abuse	NR	NR	NR	NR
WHO./2013 [18]	World Health Organization (WHO)	Governmental funding	Pregnant women actively using tobacco and pregnant women exposed to second-hand smoking	NA	Systematic review	**5 databases** (Cochrane; Database; Medline; the Campbell Collaboration Library of systematic reviews; CDSR)	NR	2010 – 2012
Kelly et al./2013 [23]	National Institute for Health and Care Excellence (NICE)	NR	People in acute, maternity and mental health services	NA	Systematic review	19 databases (AMED; ASSIA; British Nursing Index; CINAHL; Cochrane Central Register of Controlled Trials; CDSR; DARE; HTA; EMBASE; EPPI Centre TRoPHI HMIC; Medline; UK Clinical Research Network Portfolio Database; PsycINFO; Sociological Abstracts; Social Policy and Practice; Web of Knowledge; CDC Smoking & Health Resource Library database; Specialist (public health) systematic review registers; EPPI Centre; DoPHER of Health Evidence) and organizations’ websites	English language	1990–2012
Zyl-Smit et al./2013 [32]	South African Thoracic Society	None	All smokers	Pregnancy; adolescents; pediatrics; tuberculosis; HIV; mental Illness; in-hospital	Review of existing guidelines	**1 database** (PubMed) and organizations’ websites	NR	NR
McRobbie et al./2014 [24]	Ministry of Health led by The University of Auckland’s Clinical Trials Research Unit	NR	All smokers	Māori; Pacific people; Asian people; pregnant and breastfeeding women; children and young people; hospitalised and preoperative patients; people who use mental health services; users of addiction treatment services; people who make repeated quit attempts	Systematic review	**7 databases** (MEDLINE; CDSR; the Cochrane Controlled Trials Register (CENTRAL); DARE; AMED; EMBASE; PsycINFO)	English	2002 – 2006
NCSCT/2014 [25]	National Center for Smoking Cessation and Training (NCSCT)	NR	All adult smokers	Pregnancy; mental illnesses; substance misuse; prisoners; black and minority ethnic groups; children and young people	Systematic review	NR	NR	NR
Siu et al./2015 [34]	U.S. Preventive Services Task Force (USPSTF)	Agency for Healthcare Research and Quality	All adult smokers	Pregnant women	Review of systematic reviews and meta-analysis	**5 databases** (PubMed, PsycInfo; DARE; CDSR, and the Centre for Reviews and Dissemination Health Technology Assessment) organizations’ websites and experts	English	2009–2014
Batra A et al./2016 [33]	German Society for Psychiatry, Psychotherapy, Psychosomatic, and Neurology (DGPPN) as well as the German Society for Addiction Research and Addiction Therapy (DG-Sucht)	Interest-free donations, Central Institute of Mental Health in Mannheim and the Section of AddictionResearch and Addiction Medicine	All tobacco users	Adolescents; women, pregnant women; hospitalized; pre-operative; chronic obstructive pulmonary disease; cardiovascular diseases; mental illnesses	Systematic review	NR	NR	NR
Al-Katheer/2016 [26]	Ministry of Public Health of Qatar (MOPH)	NR	All smokers	Adolescents pregnant and breastfeeding women	Systematic review	NR	English	NR
Nik Mohamed et al./2016 [19]	Ministry of Health Malaysia and Malaysian Academy of Pharmacy	Ministry of health Malaysia and Malaysian Academy of Pharmacy	All adult smokers	Female smokers, pregnant and lactating women; hospitalized smokers; mental illnesses; substance use disorders; children and adolescents; elderly;	Systematic review	**4 databases** (Guideline international network; Medline; PubMed; CDSR)	English	2001 – 2016
Shields, et al./2017 [27]	NCCN – National Comprehensive Cancer Network	NR	Adult smokers with cancer	NA	Systematic review of primary literature	**1 database** (PubMed) and committee meetings	English	2015 – 2016
Schayck et al./2017 [28]	International Primary Care Respiratory Group	NR	All smokers	Pregnancy; adolescents; pediatrics, tuberculosis; HIV; mental illness; respiratory disease; cardiovascular disease	NR	NR	NR	NR
Hopkins et al./2018 [29]	National Institute for Health and Care Excellence (NICE)	NR	All adult smokers	Adolescents	Systematic Reviews	**18 databases** (CDSR; HMIC; TRIP; Applied Social Sciences Index & Abstracts; ProQuest; Cochrane Central Register of Controlled Trials; EMBASE; MedLine; NHS Economic Evaluation Database; Social Policy and Practice; Campbell Collaboration Library; DARE; Database of Promoting Health Effectiveness Reviews; Health Evidence; National Institute for Health Research Journals Library; Prospective Register of Systematic Reviews)	English	Database inception – 2016

*NA* not applicable, *NR* not reported

The majority of the guidelines (n = 14) [12, 15, 16, 18, 19, 21, 23, 27, 29–32, 34, 35] reported implementing a systematic search for primary literature through multiple resources and databases. The NICE guidelines utilized the highest number of databases and organizations’ websites compared to other guidelines [21, 23, 29]. The NCCN guideline searched only one database in addition to consensus meetings [27]. Other CPGs evaluated did not report the sources of the evidence used [13, 14, 17, 20, 22, 24–26, 28, 33]. Other characteristics of the reviewed CPGs are provided in Table 2.

**Fig. 2:**
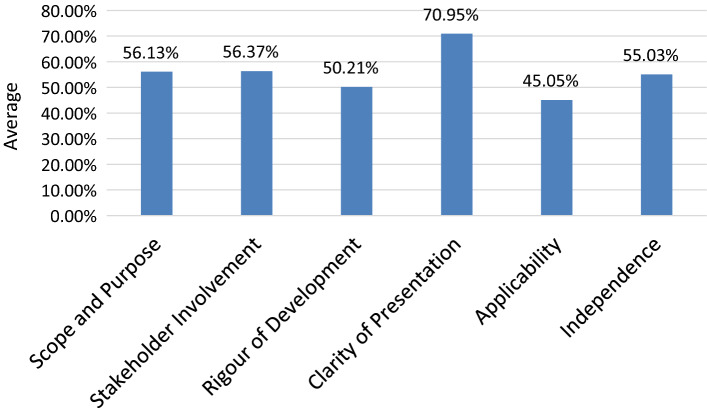
The AGREE II average score of included guidelines

The quality assessment of the 24 included guidelines is provided in Table 3 Seven guidelines [18, 19, 21, 23, 29, 34, 35] were judged to be of high quality with an overall score of > 70% based on the AGREE II instrument. The NICE guideline [29] had the highest AGREE II quality score, while the guidelines by the Japanese Circulation Society (JCS) Joint Working Group and the National Center for Smoking Cessation and Training (NCSCT) had the lowest AGREE II quality scores [22, 25]. Regarding the overall scores of guidelines on the domains of the AGREE II instrument, the highest scores were for Domain 4 (clarity of presentation) (70.95%) and Domain 2 (Stakeholders’ involvement) (56.37%). The domain with the lowest average score was Domain 5 (Applicability) (45.05%) as shown in Fig. 2.

**Table 3:** AGREE II quality of included tobacco dependence treatment guidelines

Author/year	Domain 1 *Scope and purpose*	Domain 2 *Stakeholder involvement*	Domain 3 *Rigour of development*	Domain 4 *Clarity of presentation*	Domain 5 *Applicability*	Domain 6 *Independence*	Overall	Recom.	ICC score (CI)
`Weel/2006 [30]	52.78	72.22	56.25	61.11	58.33	58.33	59.84	YM	0.827 (0.600–0.926)
Tønnesen et al./2007 [20]	63.89	44.44	15.63	80.56	41.67	0.00	41.03	YM	0.975 (0.942–0.990)
Fiore et al./2008 [35]	50.00	94.44	53.13	83.33	70.83	83.33	72.51	Yes	0.886 (0.729–0.952)
Reis et al./2008 [12]	50.00	36.11	65.63	58.33	27.08	54.17	48.55	YM	0.933 (0.842–0.971)
Schayck et al./2008 [13]	50.00	58.33	10.42	52.78	60.42	87.50	53.24	YM	0.927 (0.799–0.971)
Kelly et al./2010 [21]	88.89	100.00	94.79	80.56	66.67	25.00	75.98	Yes	0.813 (0.555–0.921)
Zwar et al./2011 [14]	44.44	52.78	25.00	63.89	45.83	50.00	46.99	YM	0.944 (0.869–0.976)
Leone F et al./2012 [15]	77.78	69.44	65.63	72.22	29.17	100	69.04	YM	0.809 (0.545–0.920)
Lingford-Hughes et al./2012 [16]	41.67	30.56	36.46	52.78	27.08	33.33	36.98	NA	0.789 (0.450–0.914)
Murohara et al./2012 [22]	47.22	36.11	15.63	41.67	35.42	0.00	29.34	NA	0.690 (0.264–0.869)
Salby et al./2012 [31]	52.78	47.22	64.58	83.33	43.75	87.50	63.19	YM	0.960 (0.907–0.983)
Chan K et al./2013 [17]	44.44	50.00	13.54	80.56	20.83	4.17	35.59	NA	0.894 (0.670–0.960)
WHO./2013 [18]	91.67	77.78	76.04	66.67	66.67	70.83	74.94	Yes	0.269 (− 0.712–0.689)
Kelly et al./2013 [23]	75.00	86.11	89.58	75.00	68.75	41.67	72.69	YM	0.221 (− 0.430–0.624)
Zyl-Smit et al./2013 [32]	11.11	38.89	28.13	72.22	6.25	95.83	42.07	NA	0.883 (0.725–0.951)
McRobbie et al./2014 [24]	41.67	52.78	61.46	80.56	56.25	50.00	57.12	YM	0.832 (0.444–0.938)
NCSCT/2014 [25]	19.44	5.56	16.67	63.89	70.83	4.17	29.40	No	0.957 (0.899–0.982)
Siu et al./2015 [34]	91.67	55.56	81.25	77.78	47.92	83.33	72.92	YM	0.885 (0.727–0.951)
Batra A et al./2016 [33]	27.78	13.89	20.83	66.67	27.08	83.33	39.93	NA	0.820 (0.583–0.923)
Al-Katheer/2016 [26]	83.33	50.00	38.54	69.44	6.25	0.00	41.96	YM	0.934 (0.826–0.973)
Nik Mohamed et al./2016 [19]	69.44	83.33	83.33	77.78	64.58	83.33	76.97	Yes	0.782 (0.465–0.909)
Shields, et al./2017 [27]	75.00	58.33	77.08	77.78	47.92	91.67	71.30	Yes	0.893 (0.745–0.955)
Schayck et al./2017 [28]	16.67	41.67	18.75	77.78	10.42	50.00	35.88	No	0.925 (0.798–0.970)
Hopkins et al./2018 [29]	80.56	97.22	96.88	86.11	81.25	83.33	87.56	Yes	0.773 (0.470–0.903)
Overall	56.13	56.37	50.21	70.95	45.05	55.03	55.63		

*Recom* recommendation; *YM* yes with modifications; *NA* no agreement; *ICC* interclass correlation coefficient; *CI* confidence interval

Considering the overall recommendations, two guidelines [25, 28] were not recommended, while six were recommended with no modifications [18, 19, 21, 27, 29, 35].

The ICC was calculated for each guideline and it reflected a very strong agreement between the reviewers, except for two guidelines [18, 23]. The average ICC score across all guidelines was 0.817 (range 0.221–0.975).

## Discussion

Tobacco use is one of the major public health threats worldwide. It is a major risk factor for cardiovascular diseases, respiratory diseases, cancers, and deaths [36]. In the last several years, several efforts have been exerted to reduce the burden of this epidemic. Despite these, a systematic and an organized approach for the treatment of tobacco use dependence is needed in different healthcare settings. Several CPGs for the treatment of tobacco use are developed and available for potential clinical use. However, data regarding the quality, rigour of development, and applicability of these guidelines are scarce. This review attempted to identify and appraise existing CPGs for tobacco cessation.

Twenty-four CPGs for the treatment tobacco use dependence met the eligibility criteria for the study and were assessed using AGREE II criteria. There was a great variability between the different guidelines in terms of their quality based on items and domains assessment. Seven guidelines were considered of high quality with an overall score of > 70% [18, 19, 21, 23, 29, 34, 35]. The guideline with the highest overall ranking score (87.56%) was the NICE guideline for stop smoking interventions and services [29]. While this guideline excelled in all domains of the Agree II criteria as compared to other guidelines, it is plausible that the developer had a superior reporting system. It is worth noting that before implementing any of the guideline recommendations, it is important to see the adaptability of these recommendations to the context of the practice setting. The guidelines with the lowest overall quality scored had consistently low scores across all domains, especially in stakeholder involvement, rigor of development, and editorial independence domains.

The domains with the lowest quality appraisal scores were ‘rigour of development’ with an average score of 50.21% and ‘applicability’ with an average score of 45.05%. These findings are consistent with previous studies’ findings [37–39]. There was agreement on only six guidelines to be recommended for use without modifications [18, 19, 21, 27, 29, 35], while 11 guidelines were recommended for use in practice with modifications [12–15, 20, 23, 24, 26, 30, 31, 34].

On the ‘rigour of development’ domain, which is of utmost importance for the quality of guidelines, only two guidelines scored over 90%. They are the NICE guidelines for “smoking: stopping in pregnancy childbirth” and NICE guideline for “stop smoking interventions” [21, 29]. Of the 24 guidelines, 11 scored less than 50% on this domain [13, 14, 16, 17, 20, 22, 25, 26, 28, 32, 33]. Under the “rigour of development” domain in the AGREE II tool, there are eight items: use of systematic methods to search for evidence, description of criteria used for selecting the evidence, description of strengths and limitations of evidence body, description of methods used for formulating recommendations, consideration of benefits, side effects, and risks in formulating recommendations, having an explicit link between recommendations and supporting evidence, review of the guideline by experts prior to its publication and provision of a procedure for updating the guideline [11]. The items with the lowest scores were in relation to the description of criteria used for selecting the evidence, description of the methods used for formulating guideline recommendations and provision of a procedure for updating the guideline. For instance, 12 guidelines did not explicitly describe the criteria that they have used for including/excluding evidence [13, 14, 16, 17, 20, 22, 24–26, 28, 32, 33], 11 guidelines did not describe what methods or systems were adopted to reach the final guideline recommendations and decisions [12–14, 17, 20, 22, 25, 26, 28, 32, 33], and 15 did not state the procedure for updating the guidelines with the latest research evidence [13–17, 20, 22, 24–26, 28, 30, 32, 33, 35]. On the other hand, the majority of the guidelines had explicitly added the link between the guideline recommendations and the research evidence based on which the guidelines have made their recommendations. In addition, in most instances, guidelines summarized their recommendations in tables along with the strength ratings of the evidence. Having clinical guidelines of high methodological rigor is crucial [8, 40]. There is a need to develop tobacco cessation guidelines while applying rigorous methodologic strategies. Without describing the exact criteria and methods used to generate the evidence, the users of guidelines would not be able to decide whether the recommendations are built on robust or weak evidence. Furthermore, guidelines should include a system to monitor the updates in evidence to ensure that recommendations are pertinent and timely [41].

In relation to the applicability of guidelines, the highest score obtained was for NICE guidelines for stop smoking interventions with a score of 81.25% [29]. Of the 24 guidelines assessed, 14 guidelines scored less than 50% [12, 14–17, 20, 22, 26–28, 31–34]. This domain is largely based on the availability of implementation tools, presence of cost analyses, and resource descriptions required for implementation. In many cases, these considerations may not be fully understood before publication and may not be applicable to all settings where the guideline may be implemented. It is likely, for example, that costs and resources of implementation may differ between institutions, healthcare settings, cities, jurisdictions, and countries [11]. The item with the lowest score under this domain was for the resource implications of applying the recommendations. Eleven guidelines did not identify the resources that are required to apply the recommendations [14, 16, 17, 19, 22, 26, 28, 31–34]. These recourses could be financial, human or physical resources. The item with the second lowest score was for inclusion of monitoring and/or auditing criteria for guideline implementation [12, 15, 16, 21, 22, 26, 28, 31, 32]. Furthermore, 6 guidelines scored poorly on the item related to the provision of advice and/or tools on how to implement recommendations in practice [12, 16, 20, 26, 28, 32]. Guidelines are not self-implementable and should be adapted to the context of the setting where they are going to be applied taking into consideration the setting’s cultural, financial and environmental factors. Evidence suggests that it is expected that clinicians and patients would benefit from guidelines containing application tools [42]. These tools could overcome many of the patient, provider, institutional and system-level barriers that could face guideline implementation. Guidelines should also include explicit criteria that originate from the main guideline recommendations to help monitoring and measuring the application of the guideline recommendations.

The guidelines that scored the highest on the domain of “scope and purpose” were WHO recommendations for the prevention and management of tobacco use and second-hand smoke exposure in pregnancy [18], NICE guideline for stopping smoking in pregnancy and after childbirth [21], and the U.S. Preventive Services Task Force Recommendation Statement [34]. These guidelines explicitly stated the objectives of the guidelines, and the patient population to whom the guidelines can be applied. While four out of 24 guidelines did not clearly describe the scope and objectives of the guidelines, their benefits, and their outcomes [24, 28, 32, 33], and targeted population [25, 28, 33, 35].

In terms of “stakeholder involvement” domain, the overall average score for the domain was 56.37%. The top performing guidelines for this domain were the Clinical Practice Guideline for Treating Tobacco Use and Dependence by the U.S. Public Health Service [35] with a score of 94.44%, NICE guidelines for stopping smoking in pregnancy and after childbirth [21] with a score of 100%, and NICE Stop smoking interventions and services [29] with a score of 97.22%. On the other hand, 10 guidelines scored less than 50% on this domain; for these guidelines, information about the stakeholder(s) involved in developing the guideline was not clear. In addition, the opinions, experiences and expectations of the target population or patients were not sought and the target users of the guideline were not defined. It is strongly recommended that the stakeholders’ opinions especially of patients would be sought when developing guideline recommendations. Involvement of patients in the decision-making process is associated with improved application of guidelines and better health outcomes [43].

In terms of clarity of presentation, the key recommendations in 15 out of the 24 guidelines were clearly either presented as a separate table or textually embedded within the guidelines. Although important for readability and usability of the guideline, it could be argued that other domains (e.g. rigour of development) may be of greater importance to guideline quality. As for editorial independence, to avoid any potential for bias, guideline developers should demonstrate that the views of the funding body have not influenced the content of the guideline and should state any conflict of interest they may have. Eight guidelines did report these two items, respectively [17, 20, 22, 24–26, 28] and [14, 16, 17, 20–22, 25, 26].

This study has implications for practice including pharmacy practice and future research. In terms of practice, users of guidelines, especially clinicians and pharmacists, should be aware of the commonly identified issues raised by this study, in order to best assess the appropriateness of adapting guidelines in practice. Users including pharmacists should also be aware of specific flaws and limitations of any guideline, specifically if these occur in domains of great importance to their population (e.g. patient preferences vs. inclusion of implementation tools). Moreover, pharmacy organizations should carefully appraise the quality of smoking guidelines before endorsement. From research perspective, the barriers to creating guidelines that are deemed of high quality by the AGREE II criteria should be explored with respect to smoking cessation. It could be possible, for example that the quality of evidence available as a whole is not at the same level as other clinical conditions. In such cases, domain scores will likely be inevitably lower and beyond the control of the guideline developer. Future research should focus on identifying how the use of AGREE II criteria in guidelines creation and reporting may influence guideline development, guideline implementation by practitioners, and any effects on patient care decisions.

This study has some limitations some of which are inherent to any systematic review. The literature search may not have identified all available guidelines on tobacco dependence treatment. However, the extensiveness of the search and inclusion of supplementary search strategy in addition to searching electronic databases might have resulted in identifying all major guideline that were available in the published literature. The second limitation is that we only included guidelines published in English. Therefore, guidelines published in other languages were excluded and not assessed. Furthermore, as per the AGREE II guidelines, it is preferred to have four reviewers per guideline; however, due to the small number of study investigators, only two investigators independently reviewed each guideline. This may not be a limitation, however, as the agreement between raters was high. Our findings were consistent with previous studies and this likely reflects the robustness of the AGREE II tool.

## Conclusion

In conclusion, the systematic search yielded 24 clinical practice guidelines that targeted tobacco cessation. Seven guidelines were considered of high quality with an overall score of over 70% based on the AGREE II instrument. Clarity of presentation was the main area of strength in these guidelines. However, the rigour of development and applicability were the major weaknesses in these guidelines. There is a need to improve the guideline development process and reporting in the field of tobacco cessation. Future developers of guidelines should develop guidelines in line with the AGREE II domains and items [11]. Description of the criteria used for selecting evidence and of the methods used for formulating guideline recommendations and explanation of the process for updating guidelines should be included. Seeking the patients’ opinions and expectations and inclusion of application tools for guidelines’ implementation to the daily healthcare practice should also be considered to improve the quality of guidelines.

## Electronic supplementary material

Below is the link to the electronic supplementary material.

Supplementary material 1 (PNG 8 kb)

Supplementary material 2 (PNG 18 kb)
