# Supplementary figures and images for: Critical appraisal of tobacco dependence treatment guidelines
Source: Int J Clin Pharm. 2020 Sep 8;43(1):85–100. doi: 10.1007/s11096-020-01110-4 (PMC7878272; doi:10.1007/s11096-020-01110-4)

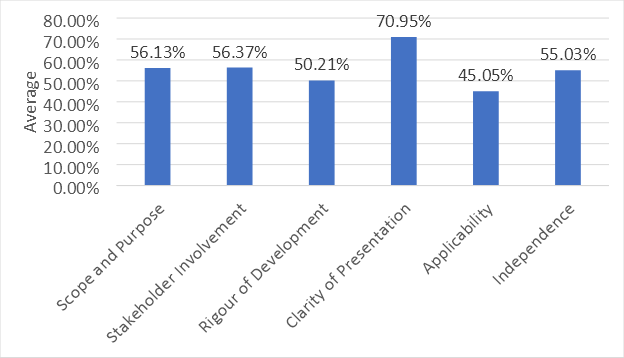

Supplement: Supplementary file 1 — Supplementary material 1 (PNG 8 kb) [file 11096_2020_1110_MOESM1_ESM.png]

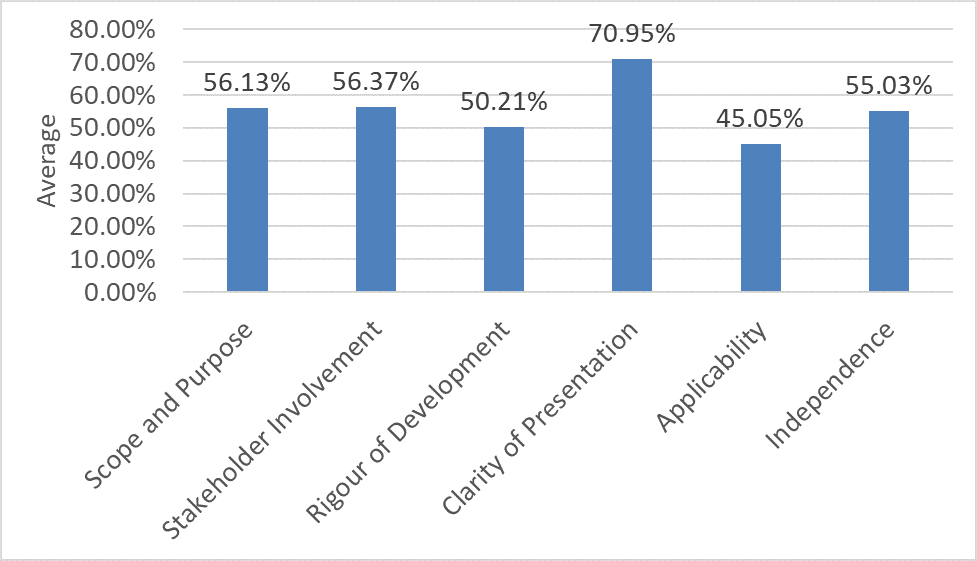

Supplement: Supplementary file 2 — Supplementary material 2 (PNG 18 kb) [file 11096_2020_1110_MOESM2_ESM.png]
